# Eyes on dialysis: acute ocular changes in adults with stage 5 chronic kidney disease before and after a single maintenance hemodialysis session

**DOI:** 10.25122/jml-2026-0090

**Published:** 2026-09

**Authors:** Lakkshya Bhramaresh Sharma, Abha Gahlot, Yamini Patial, Jawahar Lal Goyal, Bheem Raj Gupta

**Affiliations:** 1 Department of Ophthalmology, School of Medical Sciences and Research, Sharda University, Greater Noida, Uttar Pradesh, India; 2 Department of Ophthalmology, Sri Sadguru Seva Sangh Trust, Janki Kund, Chitrakoot, Madhya Pradesh, India; 3 Department of Ophthalmology, All India Institute of Medical Sciences (AIIMS), Raipur, Chhattisgarh, India

**Keywords:** renal insufficiency, chronic, renal dialysis, tomography, optical coherence, dry eye syndromes, macula lutea, visual acuity, BCVA, best-corrected visual acuity, CI, confidence interval, CKD, chronic kidney disease, CMT, central macular thickness, eGFR, estimated glomerular filtration rate, ESRD, end-stage renal disease, ETDRS, Early Treatment Diabetic Retinopathy Study, HD, hemodialysis, IOP, intraocular pressure, LogMAR, logarithm of the minimum angle of resolution, OCT, optical coherence tomography, RNFL, retinal nerve fiber layer, SD, standard deviation, TBUT, tear break-up time, UCVA, uncorrected visual acuity

## Abstract

Adults with stage 5 chronic kidney disease (CKD) receiving maintenance hemodialysis (HD) may experience acute ocular changes related to rapid osmotic and fluid shifts during treatment. Spectral-domain optical coherence tomography (OCT) provides high-resolution structural imaging of the retina and optic nerve head, and ocular surface tests can detect concurrent tear film disturbance. This study aimed to characterize the impact of a single HD session on OCT-derived metrics and ocular surface parameters in adults with stage 5 CKD. In this observational study, 50 adults with stage 5 CKD on maintenance HD for at least 3 months underwent standardized ophthalmic assessment immediately before and within 30 minutes after a single 4-hour HD session. Assessments included uncorrected and best-corrected visual acuity (UCVA, BCVA), tear break-up time (TBUT), Schirmer test I, and spectral-domain OCT-derived central macular thickness (CMT) and peripapillary retinal nerve fiber layer (RNFL) thickness. HD was associated with statistically significant worsening in UCVA and BCVA (*P* < 0.001). TBUT and Schirmer test I values declined significantly in both eyes (*P* < 0.001), and CMT was significantly reduced (*P* < 0.001). Peripapillary RNFL thickness showed a small but statistically significant reduction after HD (*P* < 0.001). A single HD session induces measurable ocular alterations in adults with stage 5 CKD, characterized by worsening visual acuity with concurrent deterioration in tear film stability and macular thinning, and a small absolute reduction in RNFL thickness. These findings support routine ophthalmic surveillance and integrated nephrology–ophthalmology care to reduce ocular morbidity in patients receiving maintenance HD.

## INTRODUCTION

Chronic kidney disease (CKD) is an escalating global public-health and socioeconomic challenge that affects an estimated 850 million people worldwide; in 2023, approximately 788 million adults were living with CKD and 4.6 million required kidney replacement therapy, including dialysis or transplantation [1,2]. This burden is particularly substantial in developing countries such as India, where demographic transition, increasing life expectancy, and the growing prevalence of diabetes and hypertension have contributed to the rising incidence of advanced CKD. Patients who progress to stage 5 CKD and require maintenance hemodialysis (HD) constitute a particularly vulnerable subgroup, with a high burden of cardiometabolic comorbidities and microvascular complications that may extend beyond the kidney to involve ocular structures.

Adults with stage 5 CKD receiving maintenance HD are exposed to rapid intravascular, osmotic, and metabolic shifts during each dialysis session, and these changes may acutely affect ocular surface homeostasis and posterior segment structure. Although some reports describe post-HD gains in visual acuity attributable to regression of macular edema in diabetic cohorts, the preponderance of data suggests exacerbation of anterior segment dysfunction, typically reflected by shortened tear break-up time (TBUT) and reduced Schirmer test I values, which can subtly degrade visual acuity, or at least the quality of its assessment, after a session [3–5]. Intraocular pressure (IOP) shows highly variable intradialytic behavior, with studies documenting an increase [6–8], a decrease [9], or negligible net change [10], while posterior segment responses are likewise heterogeneous, with reported reductions in retinal nerve fiber layer (RNFL) thickness and central macular thickness (CMT) in some series and choroidal thickening or relative structural stability in others [5,11].

Optical coherence tomography (OCT) provides high-resolution, noninvasive structural imaging of the retina and optic nerve head, enabling precise quantification of parameters such as CMT and peripapillary RNFL thickness in this high-risk population. Retinal microvasculopathy has been linked to CKD, diabetes, and hypertension, and structural OCT has demonstrated associations between reduced retinal vessel caliber and diminished RNFL thickness; however, direct visualization of the retinal microvasculature requires OCT angiography, which is distinct from the structural OCT used in many studies [3,12].

This discordant literature, particularly in the context of stage 5 CKD on maintenance HD, largely reflects methodological heterogeneity, including differences in HD vintage and session duration, comorbidity burden, imaging platforms (time-domain versus spectral-domain OCT), and the temporal relationship between dialysis and ophthalmic evaluation. Against this background, the present prospective observational study examined alterations in spectral-domain OCT-derived CMT and peripapillary RNFL thickness before and after HD, together with ocular surface metrics (best-corrected visual acuity [BCVA], TBUT, and Schirmer test I), in adults with stage 5 CKD on maintenance HD undergoing a single standardized 4-hour session. The objective was to provide an integrated same-session analysis of visual acuity, ocular surface status, and structural OCT metrics under a uniform examination protocol. This approach is clinically relevant because transient dialysis-related ocular changes in stage 5 CKD may confound routine ophthalmic assessment and are particularly important in patients with diabetes or other coexisting retinal risk factors, thereby informing the optimal timing and breadth of nephrology–ophthalmology care pathways.

## MATERIAL AND METHODS

### Study design and setting

This hospital-based prospective observational study was conducted in the hemodialysis unit of a tertiary care center in India between June 2023 and January 2025. Adults aged 18 to 70 years with stage 5 CKD, defined by an estimated glomerular filtration rate (eGFR) < 15 mL/min/1.73 m^2^ and receiving maintenance HD for at least 3 months, were eligible.

### Sampling strategy

Consecutive eligible patients attending the maintenance HD unit during the study period were approached for inclusion. All patients who met the inclusion criteria and provided written informed consent were enrolled until the target sample size of 50 participants was reached. No randomization or matching was performed; dialysis prescription and ultrafiltration were determined independently by the treating nephrologist.

### Inclusion and exclusion criteria

Patients with BCVA better than 6/18 (0.48 LogMAR) and sufficiently clear ocular media to permit reliable OCT imaging were included. Exclusion criteria comprised a history of neurodegenerative disease or intracranial pathology, high refractive error (greater than ±5.00 diopters sphere or ±2.00 diopters cylinder), glaucoma, suboptimal OCT image quality, poor fixation (e.g., nystagmus or poor cooperation), prior posterior segment surgery, and retinal disorders such as retinal vein occlusion, age-related macular degeneration, macular hole, or epiretinal membrane, because these independently affect optic nerve head structure and RNFL thickness and could therefore confound interpretation of acute dialysis-related changes in OCT-derived neuroretinal parameters. Patients with corneal scarring, uveitis, prior peritoneal dialysis, or previous kidney transplantation were also excluded.

### Sample size

Sample size was determined using Slovin’s formula. The incidence was derived from the nephrology department’s registry of new cases, an average of 5 patients per month (90 over 18 months). With a 10% margin of error, Slovin’s formula yielded a minimum of 47 participants; we therefore prospectively enrolled 50 patients to ensure adequate precision.

### Study procedure and ocular assessment

A standardized pre-HD examination sequence was used. Distance uncorrected visual acuity (UCVA) and BCVA were measured at 6 m using Snellen charts and converted to LogMAR notation; pinhole testing was performed when acuity was 6/12 (0.30 LogMAR) or worse. TBUT was assessed by instilling 5 µL of 1% fluorescein and observing the tear film under cobalt blue illumination with a ring filter; five consecutive readings per eye were obtained and averaged to improve reliability. Schirmer test I was performed with standard filter paper strips for 5 minutes without topical anesthesia under controlled ambient humidity (40–60%). Posterior segment assessment used the REVO FC spectral-domain OCT system (Optopol Technology Sp. z o.o., Zawiercie, Poland). The macular cube protocol (512 × 128 A-scans, Early Treatment Diabetic Retinopathy Study [ETDRS] grid) automatically quantified CMT within the central 1-mm foveal zone, while the fast RNFL protocol acquired 3.4-mm peripapillary circular scans (256 A-scans) centered on the optic disc.

Quality assurance criteria required a minimum OCT signal strength of 7/10; scans showing motion artifacts, decentration, or segmentation errors were excluded from analysis. Post-HD evaluations replicated the pre-HD protocol in full and were performed within 30 minutes of completing a single 4-hour HD session (heparinized dialyzer, bicarbonate-based dialysate, and individualized ultrafiltration as per nephrologist prescription). All ocular measurements were obtained by a single masked examiner to minimize interobserver variability and ensure procedural consistency.

### Statistical analysis

Data were entered and analyzed using IBM SPSS Statistics version 25.0 (IBM Corp., Armonk, NY, USA). Normality was evaluated with the Shapiro–Wilk test; paired *t* tests or Wilcoxon signed-rank tests were applied as appropriate. Effect sizes were expressed as Cohen’s *d* (0.2, small; 0.5, medium; 0.8, large; ≥1.2, very large). The study achieved 92% statistical power (α = 0.05, effect size = 0.8), and statistical significance was defined as *P* < 0.05. Exact *P* values are reported for all comparisons except values below 0.001, which are expressed as *P* < 0.001.

The primary analyses compared within-subject changes in ocular parameters before and after HD without covariate adjustment. Recorded systemic comorbidities are summarized in Table 1.

**Table 1:** Baseline characteristics of the participants

Characteristic (*n* = 50)	Value
Age, years, mean ± SD	55.4 ± 7.7
Age range, years	39–69
Female sex, *n* (%)	26 (52.0)
Male sex, *n* (%)	24 (48.0)
Diabetes mellitus, *n* (%)	36 (72.0)
Non-diabetic CKD, *n* (%)	14 (28.0)

Data are presented as mean ± SD or *n* (%). CKD, chronic kidney disease; *n*, number of participants; SD, standard deviation.

## RESULTS

Data from 50 patients were analyzed, with both eyes included for each patient, yielding a total of 100 eyes that completed the HD session. The mean age of the participants was 55.4 ± 7.7 years (range, 39–69 years). There were 26 women (52.0%) and 24 men (48.0%). Diabetes mellitus was present in 36 participants (72.0%), while 14 participants (28.0%) had non-diabetic CKD. Detailed baseline characteristics are presented in Table 1.

Visual acuity worsened after HD, with both UCVA and BCVA showing statistically significant declines in both eyes: UCVA increased by 0.07 LogMAR (95% confidence interval [CI], 0.04–0.09) in the right eye and 0.09 LogMAR (95% CI, 0.07–0.12) in the left eye, while BCVA increased by 0.07 LogMAR (95% CI, 0.04–0.10) in the right eye and 0.06 LogMAR (95% CI, 0.04–0.09) in the left eye (all *P* < 0.001) (Table 2). Ocular surface parameters also deteriorated: TBUT decreased by 3.48 seconds (95% CI, −4.14 to −2.82) and 3.44 seconds (95% CI, −3.78 to −3.10), and Schirmer test I decreased by 3.26 mm (95% CI, −3.58 to −2.94) and 2.24 mm (95% CI, −2.44 to −2.04) in the right and left eyes, respectively (*P* < 0.001), indicating reduced tear film stability and aqueous secretion (Table 3).

**Table 2:** Uncorrected and best-corrected visual acuity before and after a single hemodialysis session

Parameter (LogMAR)	Before HD (mean ± SD)	After HD (mean ± SD)	Mean change (after − before)	95% CI of the difference	*P*
Right eye UCVA	0.13 ± 0.11	0.20 ± 0.10	+0.07	+0.04 to +0.09	**< 0.001**
Right eye BCVA	0.02 ± 0.07	0.09 ± 0.10	+0.07	+0.04 to +0.10	**< 0.001**
Left eye UCVA	0.08 ± 0.09	0.17 ± 0.09	+0.09	+0.07 to +0.12	**< 0.001**
Left eye BCVA	0.02 ± 0.06	0.09 ± 0.09	+0.07	+0.04 to +0.09	**< 0.001**

Data are presented as mean ± SD for 50 participants per eye. Visual acuity is expressed in LogMAR, so higher values indicate worse acuity and a positive mean change indicates worsening. Comparisons were made with the paired t-test. Bold *P* values indicate statistical significance (*P* < 0.05). BCVA, best-corrected visual acuity; CI, confidence interval; HD, hemodialysis; LogMAR, logarithm of the minimum angle of resolution; SD, standard deviation; UCVA, uncorrected visual acuity.

**Table 3:** Tear break-up time, Schirmer test I, central macular thickness and peripapillary retinal nerve fiber layer thickness before and after a single hemodialysis session

Outcome	Before HD (mean ± SD)	After HD (mean ± SD)	Mean paired difference (after − before)	95% CI of the paired difference	*t (df)*	*P*
Right eye TBUT (s)	17.72 ± 2.57	14.24 ± 2.88	−3.48	−4.14 to −2.82	−10.67 (49)	**< 0.001**
Left eye TBUT (s)	17.80 ± 3.19	14.36 ± 3.31	−3.44	−3.78 to −3.10	−20.60 (49)	**< 0.001**
Right eye Schirmer test I (mm)	18.22 ± 5.85	14.96 ± 5.55	−3.26	−3.58 to −2.94	−20.23 (49)	**< 0.001**
Left eye Schirmer test I (mm)	18.66 ± 6.03	16.42 ± 5.97	−2.24	−2.44 to −2.04	−23.06 (49)	**< 0.001**
Right eye CMT (µm)	242.86 ± 11.54	218.36 ± 13.80	−24.50	−26.48 to −22.52	−24.88 (49)	**< 0.001**
Left eye CMT (µm)	242.20 ± 11.73	234.24 ± 13.13	−7.96	−9.30 to −6.62	−11.93 (49)	**< 0.001**
Right eye RNFL (µm)	88.46 ± 8.27	87.10 ± 8.28	−1.36	−1.69 to −1.03	−8.19 (49)	**< 0.001**
Left eye RNFL (µm)	89.28 ± 9.60	87.78 ± 9.55	−1.50	−1.79 to −1.21	−10.45 (49)	**< 0.001**

Data are presented as mean ± SD for 50 participants per eye. Comparisons were made with the paired *t*-test. Bold *P* values indicate statistical significance (*P* < 0.05). CI, confidence interval; CMT, central macular thickness; df, degrees of freedom; HD, hemodialysis; RNFL, retinal nerve fiber layer; SD, standard deviation; TBUT, tear break-up time.

Central macular thickness thinned significantly in both eyes after HD (right, −24.50 µm; 95% CI, −26.48 to −22.52; left, −7.96 µm; 95% CI, −9.30 to −6.62; both *P* < 0.001), while global peripapillary RNFL thickness showed small but statistically significant reductions (right, −1.36 µm; left, −1.50 µm; both *P* < 0.001).

Separate-eye analysis showed large effect sizes for UCVA in the right eye (*d* = +0.80) and left eye (*d* = +1.06), and medium effect sizes for BCVA in the right eye (*d* = +0.71) and left eye (*d* = +0.74). TBUT, Schirmer test I and CMT showed very large effect sizes in both eyes. RNFL thickness showed a large effect in the right eye (*d* = −1.16) and a very large effect in the left eye (*d* = −1.48). However, the small absolute RNFL reductions should be interpreted cautiously (Table 4; Figure 1). Violin plots depicting the full data distributions confirmed that most eyes shifted toward worse ocular status after HD, with the most pronounced distributional shifts in visual acuity, TBUT, Schirmer test I, and CMT, and a small reduction in RNFL thickness across the 50 participants (Figure 2).

**Table 4:** Effect size for each ocular parameter, separate-eye paired analysis

Parameter	Right eye, Cohen’s *d* (*n* = 50)	Left eye, Cohen’s *d* (*n* = 50)
UCVA	+0.80	+1.06
BCVA	+0.71	+0.74
TBUT	−1.51	−2.91
Schirmer test I	−2.86	−3.26
CMT	−3.52	−1.69
RNFL	−1.16	−1.48

Cohen's *d* is interpreted as 0.2, small; 0.5, medium; 0.8, large; ≥1.2, very large. Positive values indicate an increase after HD and negative values indicate a decrease; because visual acuity is expressed in LogMAR, a positive value for UCVA and BCVA indicates worsening. BCVA, best-corrected visual acuity; CMT, central macular thickness; HD, hemodialysis; RNFL, retinal nerve fiber layer; TBUT, tear break-up time; UCVA, uncorrected visual acuity.

**Figure 1:**
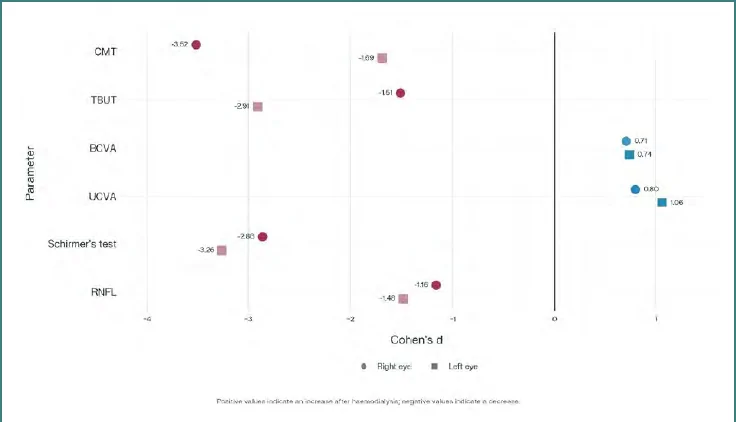
Effect sizes (Cohen's *d*) for each ocular parameter after a single hemodialysis session. Points show the standardized before- to after-hemodialysis change for 50 participants per eye, analyzed separately for right eyes (circles) and left eyes (squares). Positive values indicate an increase in LogMAR, that is, worsening visual acuity; negative values indicate a reduction in the measured parameter. BCVA, best-corrected visual acuity; CMT, central macular thickness; RNFL, retinal nerve fiber layer; TBUT, tear break-up time; UCVA, uncorrected visual acuity.

**Figure 2:**
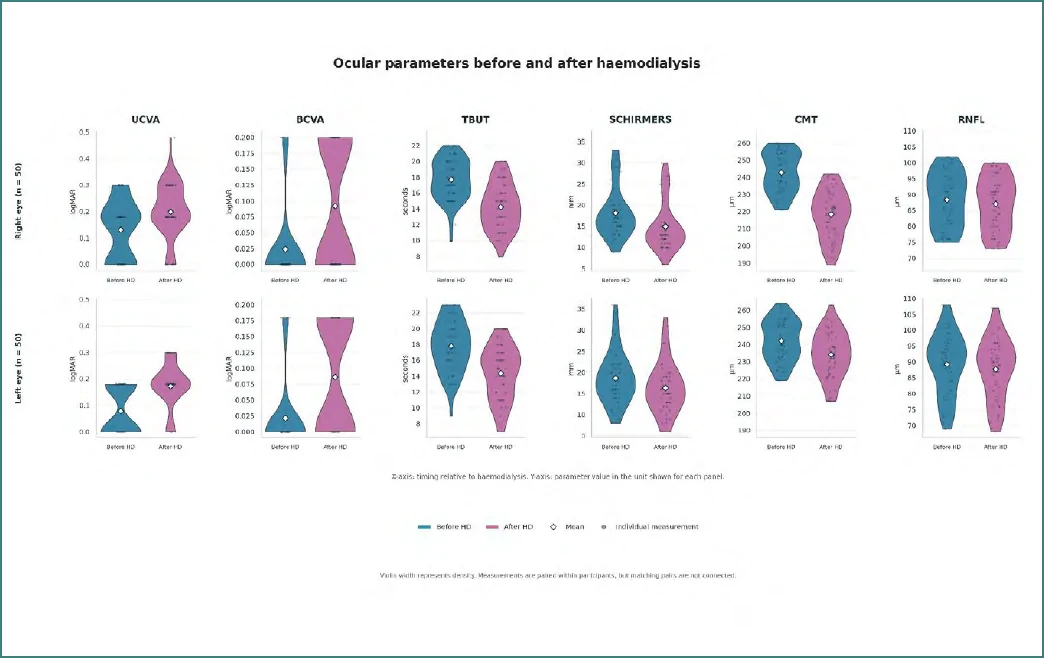
Distribution of ocular parameters before and after a single hemodialysis session. Violin plots show measurements separately for the right and left eyes of 50 participants at each time point. Blue indicates before-hemodialysis values and pink indicates after-hemodialysis values. Small dots represent individual eye measurements, and white diamonds indicate means. Violin width reflects the density of observations. Each parameter uses its own measurement scale: UCVA and BCVA in LogMAR, TBUT in seconds, Schirmer test I in mm, and CMT and RNFL thickness in µm.

## DISCUSSION

The principal findings of this study were that a single HD session in adults with stage 5 CKD was associated with transient worsening of UCVA and BCVA, significant deterioration of TBUT and Schirmer test I values, and a significant reduction in CMT, whereas global peripapillary RNFL thickness showed a small but statistically significant reduction in the immediate post-dialysis period.

In keeping with the age and sex distribution reported by Kianersi *et al*. in a larger HD cohort, the present sample represents a typical middle-aged to older adult end-stage renal disease (ESRD) population [13]. The upper age limit was selected to reduce confounding from advanced age-related ocular comorbidity that could independently influence visual acuity, tear film parameters, and OCT-derived retinal or optic nerve measurements in a single-session physiological study.

Diabetes mellitus was present in 72.0% of the cohort, with non-diabetic patients comprising 28.0%. This high diabetic burden is consistent with previous studies identifying diabetes as a leading cause of ESRD and a major driver of dialysis initiation [14,15]. Kianersi *et al*. similarly reported diabetes and hypertension as the predominant underlying conditions in HD patients [13]. The high prevalence of diabetes in our study is consistent with a multicenter Karnataka ESRD study, in which diabetes was the leading cause of CKD, followed by hypertension and chronic glomerulonephritis [16]. Manaviat *et al*. reported dry-eye syndrome in 54.3% of patients with type 2 diabetes, highlighting the burden of ocular-surface dysfunction in this population [17].Because the study was designed for within-subject before-and-after comparisons rather than subgroup analyses, the effects of HD were not adjusted for diabetes or other comorbidities, and these factors should be regarded as potential covariates in future studies using multivariate models.

In the present cohort, HD was associated with a statistically significant worsening of both UCVA and BCVA in both eyes, indicating a transient decline in visual function after a single dialysis session. This may be related in part to ocular surface deterioration, as previous studies have demonstrated post-dialysis reductions in TBUT and Schirmer test I values that can impair visual quality [4,18]; the concomitant reduction in CMT may reflect retinal fluid redistribution, although its direct contribution to the change in visual acuity remains less certain [19,20]. Jung *et al*. demonstrated significant post-dialysis reductions in TBUT, Schirmer test I values, and macular thickness, and further observed that ocular surface changes after HD affected visual acuity and examination quality [4]. This is particularly relevant to the present findings, in which reduced visual acuity occurred in parallel with significant deterioration in tear film stability, aqueous tear production, and CMT. Some authors have also suggested that refractive fluctuation during HD may reflect osmolality-driven changes in crystalline lens hydration [20–22], while Çalışkan *et al*. reported no significant dialysis-related optical changes in the cornea and lens [23]. These mechanisms are compatible with the present findings, although refraction, corneal thickness, and lens biometry were not assessed directly in this study. Accordingly, the deterioration in visual acuity should be interpreted cautiously as a multifactorial phenomenon, likely influenced by ocular surface instability, transient refractive change, and fluid redistribution rather than by a single isolated mechanism.

HD also produced significant reductions in TBUT (*P* < 0.001), Schirmer test I values (*P* < 0.001), and CMT (*P* < 0.001) in both eyes, indicating tear film instability, diminished aqueous tear production, and macular thinning. The marked bilateral decline in TBUT and Schirmer test I values after HD was consistent with the observations of Chen *et al*. [11]. Jung *et al*. further reported post-HD deterioration in ocular surface parameters, including TBUT and basal tear secretion, together with an increased keratoepitheliopathy score after a single HD session [4]. Given the high prevalence of diabetes in our cohort, diabetes-associated impairment of autonomic lacrimal regulation may have contributed to the observed post-HD reduction in TBUT and Schirmer test I values [24]; however, this association could not be examined directly in the present study.

Similarly, the significant post-HD reduction in CMT observed in both eyes may reflect acute dialysis-associated osmotic and fluid shifts affecting macular thickness. This finding is consistent with Theodossiadis *et al*., who reported significant reductions in OCT-measured macular thickness after hemodialysis in diabetic patients with ESRD, particularly in eyes with diabetic macular edema [19].

In contrast, peripapillary RNFL thickness showed a small but statistically significant reduction after HD. However, these changes were below typical OCT test–retest variability thresholds and are therefore unlikely to represent clinically meaningful acute optic nerve thinning. This differs from Emre *et al*., who reported no significant acute RNFL change after a single HD session [25], while other studies have described transient, quadrant-specific, or chronic RNFL alterations in dialysis populations [26–28]. Such discrepancies are likely related to differences in dialysis modality, duration of exposure, underlying ocular status, and imaging methodology.

In this context, transient post-HD artifacts can confound routine ophthalmic surveillance. Regular ocular surface evaluation (TBUT and Schirmer test I) may be worth embedding within HD care pathways to detect tear film instability. Periodic spectral-domain OCT imaging may assist longitudinal monitoring of macular thickness, particularly in patients with diabetes who are at heightened risk of superimposed retinopathy. Systematic tracking of visual acuity can inform counseling regarding anticipated transient change. Long-term RNFL surveillance remains prudent despite the small acute changes, especially in patients with coexistent glaucoma or optic neuropathy. Multidisciplinary nephrology–ophthalmology protocols, including integration of OCT-based screening into HD units, potentially via portable platforms, may improve early detection and management of vision-threatening pathology.

Several limitations warrant consideration. The single-center design may limit generalizability, and the focus on acute before-versus-after single-session effects precludes conclusions about chronic structural change. Intraocular pressure and ocular perfusion parameters were not collected, limiting mechanistic interpretation. Nonetheless, the prospective design, the high reproducibility of spectral-domain OCT, and the specific relevance to the stage 5 CKD HD population strengthen the validity of the findings. HD induced bilateral perturbation across the measured ocular domains, with effect sizes extending beyond statistical significance alone. Worsening of visual acuity is consistent with osmotic changes in lens hydration and with corneal fluid normalization; destabilization of the tear film reflects the well-described exacerbation of dry eye associated with HD; and reduction in CMT suggests a transient macular fluid shift. The small post-HD reduction in RNFL thickness was below typical OCT repeatability thresholds and is unlikely to represent clinically meaningful acute optic nerve injury after a single HD session.

## CONCLUSION

A single HD session was associated with measurable short-term changes across several ocular parameters in adults with stage 5 CKD, affecting tear film stability, aqueous tear production, macular thickness, and visual acuity. Visual acuity worsened transiently, ocular surface parameters deteriorated, and CMT decreased; these changes may reflect the rapid fluid and osmotic shifts that occur during dialysis, although the present study did not measure those mechanisms directly. In contrast, global peripapillary RNFL thickness showed a small statistically significant reduction immediately after dialysis; this change was below OCT repeatability thresholds and is unlikely to be clinically meaningful. Taken together, these findings indicate that the timing of ophthalmic assessment relative to dialysis warrants attention, and they support closer nephrology–ophthalmology collaboration in the care of adults with stage 5 CKD receiving maintenance HD. Confirmation in larger, multicenter cohorts with longitudinal follow-up is required before clinical recommendations can be drawn.

## Data Availability

De-identified participant-level data underlying this study, including demographic variables and pre- and post-hemodialysis measurements of visual acuity, tear break-up time, Schirmer test I, central macular thickness, and retinal nerve fiber layer thickness, are available from the corresponding author (YP) on reasonable request, subject to approval by the Institutional Ethics Committee of Sharda University and compliance with applicable participant-consent and data-protection requirements.
